# Validation of self-reported oral health among Indonesian adolescents

**DOI:** 10.1186/s12903-021-01953-x

**Published:** 2021-11-19

**Authors:** Ary Agustanti, Atik Ramadhani, Melissa Adiatman, Anton Rahardjo, Maha El Tantawi, Diah Ayu Maharani

**Affiliations:** 1 Department of Preventive and Public Health Dentistry, Faculty of Dentistry, Universitas Indonesia, Jalan Salemba No. 4, Jakarta, 10430 Indonesia; 2 Department of Pediatric Dentistry and Dental Public Health, Faculty of Dentistry, Alexandria University, Alexandria, Egypt

**Keywords:** Self-report, Oral health, Adolescents, Survey, Caries, Tooth loss, Indonesia

## Abstract

**Background and aim:**

With the recognition of health as a subjective state, self-reported oral health has been applied in many epidemiological studies. However, the validity of self-reports may vary across different age groups and socio-cultural backgrounds and by using different tools. This study aimed to assess the validity of self-reported oral health of 15-year-old Indonesian adolescents.

**Materials and methods:**

This study used data from the Indonesian National Oral Health Survey, a part of the Indonesian Basic Health Survey 2018. The study included 572 15-year-old Indonesian adolescents. We compared the presence of clinically assessed dental caries, tooth loss, and fillings following the World Health Organization Basic Health Survey method and questionnaire-based self-reported oral conditions using McNemar test. The sensitivity (Sn), specificity (Sp), and likelihood ratios (LRs) of self-reports were calculated using clinical assessment as the reference standard. The overall accuracy of self-reports in identifying the clinical condition was assessed using the area under the curve (AUC) of a receiver operating characteristic curve.

**Results:**

Self-reports significantly underestimated the clinical presence of caries (39.3% and 67.1%) and overestimated the clinical presence of tooth loss (9.3% and 4.2%) and filling (4.7% and 2.4%, *p* < 0.05). All self-reported conditions had higher Sp (at least 70.3%) than Sn (max 54.2%) and the AUC for all self-reported conditions were < 0.7. Self-reporting the presence of fillings had the highest LR+  = 11.

**Conclusions:**

Self-reporting oral health in Indonesian adolescents had low accuracy. Further studies of other methods of self-reporting are needed before they can be used to assess adolescents’ oral health in epidemiological surveys.

## Introduction

Information is needed to monitor oral health conditions and trends over time. Self-reporting of health conditions, as a measure of collecting health information, is gaining acceptance in many oral epidemiological surveys due to its convenience, lower cost compared to clinical assessment, ease of incorporation into health surveys and because it is based on one’s perception of health and disease [1–4]. Even though clinical examination serves as the reference standard in oral health surveys, self-reporting is useful in settings where limited resources and highly prevalent oral health problems are present [1]. Self-reporting of oral health can be incorporated into surveys using questionnaires where data on physical, mental, and functional health and health behaviors are collected by non-specialized personnel, thus allowing for efficient large-scale surveys [1, 2, 5]. Self-reporting of oral health conditions in population-based surveys may be a useful screening tool for the presence of oral health problems among adolescents. This may provide a cost-effective and rapid method of identifying those who need clinical evaluation and can benefit from a dental referral. Moreover, self-reported oral health expands the understanding of oral health problems among adolescents, thus serving as a valuable tool for health policymaking and public health program.

Individual perception of oral health involves a comprehensive understanding of oral disease, tissue damage, altered functional ability, pain, and aesthetics as part of psychological and psychosocial aspects [6]. Previous studies have shown that the validity of self-reported oral health can vary from promising to questionable depending on the assessed condition, tools, setting and population [1–9]. Self-reported use of dentures and number of teeth present in the oral cavity had good validity while reporting the presence of gingivitis and oral hygiene status had poor validity [7]. Assessing the validity of self-reported oral health in different populations is important because of the differences in validity among social groups and age cohorts [9]. Moreover, subjective assessment of oral health can vary based on individual beliefs, cultural background, social, educational, and environmental conditions, oral health, and awareness [4, 8].

Indonesia is the largest island nation and the 4th most populous country in the world. In 2020, its population was estimated to be about 270 million with more than 1300 diverse ethnic groups [10]. This makes it challenging to conduct epidemiological surveys using clinical examinations. In this context, self-reported oral health may serve as an efficient method for obtaining periodically updated oral health data. However, the validity of self-reporting should be assessed prior to its use. One of the age groups recommended by WHO for oral health surveys is the 15-year-old group, where permanent teeth have been exposed to the oral environment for several years [11] and because of the potentially increased risk of oral diseases in this age [12]. Assessing adolescents’ self-reported oral health provides an insight about the oral health problems in the community. This is because, during this period, adolescents are adopting new practices, attitudes, and behaviors, and have increased autonomy, which may expose them to risks affecting their oral health [13].

Only a limited number of studies are available about the accuracy of self-reporting of oral health condition among adolescents [1, 6]. This study aimed to analyze the validity of self-reporting of some oral diseases by 15-year-old Indonesian adolescents compared to clinical assessment.

## Materials and methods

Reporting of this study has been done in accordance with the STARD reporting guidelines [14]. Ethics approval was obtained from the Dental Research Ethics Committee, Faculty of Dentistry, Universitas Indonesia (Protocol Number: 030090421). This study was conducted in full accordance with the World Medical Association Declaration of Helsinki [15]. This was a secondary analysis of data from the Indonesian National Oral Health Survey (INOHS) of 2018 which was part of the Indonesian Basic Health Research (IBHR) [16, 17]. The IBHR is a national survey that is conducted every five years and covers all households in Indonesia [16]. In 2018, the IBHR included 1,017,290 persons from 34 provinces [16]. The IBHR uses a multistage systematic random cluster sampling strategy with probability proportional to size considering urban–rural distribution [17]. The first stage was based on stratification by the Wealth Index to select the census block, followed by another stage based on the education level of the head of household [16]. The INOHS 2018 included a subset of persons assessed in the IBHR who were ≥ 3 years old selected randomly from the participants in the main survey. The INOHS participants were distributed among the World Health Organization (WHO) Basic Oral Health Survey age categories [11]. A higher proportion of participants was included in the 35–44 and ≥ 60 years old age group and a lower proportion represented children and adolescents [16]. In the 15-year-old age category, 572 adolescents were recruited, and they were included in the present study.

Data were obtained using oral health examinations and questionnaires. The examinations were performed by 200 dentists who received training for calibration divided into three stages: (1) Instructional phase, where each examiner was introduced to the examination procedure and the assessment criteria; (2) Standardization phase, where each examiner is trained to apply standard operating procedures and criteria for the assessment of oral health; and (3) Calibration phase, where the measurement of the agreement between each examiner and a gold standard was carried out. Only those who achieved an acceptable agreement (Kappa > 0.6) participated in the examination [18].

The clinical examination assessed all teeth (excluding third molar) following the WHO Basic Oral Health Survey methods and criteria [11]. Any tooth with decay and a tooth that was filled but had decay were recorded as decayed, a filled tooth without caries was recorded as filled, and a tooth that was missing due to caries or other causes was recorded as tooth loss. Decay was assessed at the cavitation level using visual criteria with a blunt probe to ascertain the presence of undermined enamel if present [11]. Self-reported oral health was obtained from the participants, who were adolescents, using dichotomous answers (Yes/No) to the questions: “In the past year, have you had any problem of (a) Tooth decay, cavities or pain? (b) Missing tooth due to extraction or falling out by itself? (c) Tooth that has been filled due to cavity?” The definitions for these oral conditions were added to the question as follows:

1. *Dental caries* Cavity on the hard surface of the tooth that ranges from a small hole to a cavity that destroys the tooth
2. *Tooth loss* Missing tooth from the oral cavity
3. *Filling* Treatment for cavities performed by closing the cavity with filling material after the damaged tissue is cleaned and removed

Those who answered “Yes” to any of these questions were considered to have reported the presence of the condition. The questionnaire also assessed the socio-demographic background, including sex (male or female), residential area (urban or rural), and parental education (high, for those whose highest education was high school or higher; or low, for those whose highest education was middle school or lower).

Based on the number of teeth clinically-assessed with dental caries, two cut-off points were used to categorize the presence and absence of caries as follows [19]:

1. Caries is present when the number of decayed teeth ≥ 1, and absent when the number of decayed teeth = 0.
2. Caries is present when the number of decayed teeth > 2 and clinically absent when the number of decayed teeth = 0–2

For the presence of tooth loss and filling, only one cut-off point was set due to the low clinical representation. These conditions were considered as present when any tooth loss or filling was present and as absent when none was present based on clinical examination.

Data were analyzed using SPSS 25 (SPSS, Inc., Chicago, IL, USA). For descriptive statistics, the absolute and relative frequencies were calculated. Data were weighted to ensure that the sample was representative of the Indonesian population. The association between sex, residential area, and parental education on one side and the clinical presence of the three conditions was assessed using chi squared test. McNemar’s test was used to assess the association between the self-reported and clinically assessed condition. The statistical significance was set at *p* < 0.05.

Clinical examination and self-reported oral health were compared for diagnostic validity using Sensitivity, Specificity, Positive Likelihood Ratio (LR+), and Negative Likelihood Ratio (LR−) [20]. The LRs assess accuracy by how much self-reporting increases or decreases the pre reporting probability of the clinical condition. LRs are categorized based on the magnitude of change ranging from minimal to large difference in likelihood [21]. The farther the values of LRs from zero, the better self-reporting is at assessing the likelihood of the clinical condition. Thus, LR+ between 5 and 10 indicates moderate likelihood and LR+  > 10 indicates large likelihood of the presence of disease whereas LR− between 0.1 and 0.2 indicate moderate likelihood and LR−  < 0.1 indicates large likelihood of the absence of the clinical condition [22]. The area under curve (AUC) of a receiver operating characteristic (ROC) curve was used to quantify the overall ability of self-reporting to discriminate between the presence and absence of the clinical condition. AUC ranges from 0.5 to 1.0, with AUC = 0.5 indicating no discriminative value and 1.0 being perfect discriminative ability [23].

## Results

This study analyzed data from 572 adolescents, with slightly higher percentage of males than females (50.9% and 49.1%). Most participants lived in urban areas (55.4%) and had parents with low education (59.6%). Adolescents who visited the dentists in the past year were 14.6%. There were no statistically significant differences by sex, residential area, or parental education in all clinically assessed conditions (*p* > 0.05, Table 1).

**Table 1:** Sociodemographic characteristics of the study participants (n = 572) and association with clinically assessed oral conditions

Variables	N (%)	Dental caries n (%)	*p* Value	Tooth loss n (%)	*p* Value	Filling n (%)	*p* Value
Gender			0.053		0.476		0.380
Male	291 (50.9)	184 (63.2)		10 (3.4)		5 (1.7)	
Female	281 (49.1)	200 (71.2)		14 (5)		9 (3.2)	
Residential area			0.509		0.933		0.343
Urban	317 (55.4)	217 (68.5)		14 (4.4)		10 (3.2)	
Rural	255 (44.6)	167 (65.5)		10 (3.9)		4 (1.6)	
Parental education			0.233		1.000		0.309
High	231 (40.4)	148 (64.1)		10 (4.3)		8 (3.5)	
Low	341 (59.6)	236 (69.2)		14 (4.1)		6 (1.7)	

The most frequent oral health condition was caries (39.3% of self-reported conditions and 67.1% of clinically assessed conditions, Table 2). The presence of filling was the least self-reported (4.7%) and clinically assessed condition (2.4%). Also, 9.3% reported tooth loss and clinical assessment showed its presence in only 4.2%. There were statistically significant differences between all three self-reported and clinically assessed conditions. Compared to the clinical examinations, self-reporting underestimated the presence of dental caries but overestimated the presence of tooth loss and filling.

**Table 2:** Association between self-reported and clinically assessed oral conditions in 15-years-old Indonesian adolescents (n = 572)

Self-reports		Clinical examination		All n (%)*	*p* Value of McNemar test
		Present n (%)	Absent n (%)		
Dental caries	Yes	183 (81.3)	42 (18.7)	225 (39.3)	0.001
	No	201 (57.9)	146 (42.1)	347 (60.7)	
	All	384 (67.1)	188 (32.9)	572 (100)	
Tooth loss	Yes	6 (11.3)	47 (88.7)	53 (9.3)	0.001
	No	18 (3.5)	501 (96.5)	519 (90.7)	
	All	24 (4.2)	548 (95.8)	572 (100)	
Filling	Yes	6 (22.2)	21 (77.8)	27 (4.7)	0.026
	No	8 (1.5)	537 (98.5)	545 (95.3)	
	All	14 (2.4)	558 (97.6)	572 (100)	

*Percentage calculated from column total. All other percentages are calculated from row total

The validity of self-reporting the three oral conditions is summarized in Table 3. All self-reported conditions scored higher in specificity than sensitivity. The AUC values were < 0.7. Reporting the presence of filled teeth had the greatest AUC and the highest specificity. Reporting the presence of dental caries with the two cut-off points had the highest sensitivity and the lowest specificity. Higher cut-off points indicating the presence of more than two teeth affected with dental caries resulted in lower specificity, but higher sensitivity than the lower cut-off point. Reporting the presence of tooth loss had the lowest sensitivity.

**Table 3:** Validity of self-reporting oral health conditions relative to clinical evaluation of the 15-year-old Indonesian adolescents (n = 572)

Self-reported condition	AUC	95% CI	SN (%)	SP (%)	LR+	LR−
Dental caries						
Caries presence (Decay ≥ 1)	0.627	0.579–0.674	47.7	77.7	2.14	0.67
Caries cut off (Decay > 2)	0.623	0.575–0.670	54.2	70.3	1.82	0.65
Tooth loss	0.582	0.454–0.710	25.0	91.4	2.91	0.82
Filling	0.695	0.525–0.866	42.9	96.2	11	0.59

*AUC* area under curve, *CI* confidence interval, *SN* sensitivity, *SP* specificity, *LR+* likelihood ratio for positive value, *LR− *likelihood ratio for negative value

Self- reporting the presence of filling had LR+  = 11 and LR−  = 0.59, the highest and lowest values respectively among all conditions.

## Discussion

The findings showed differences between self-reported and clinically assessed presence of caries, tooth loss and fillings in 15-year-old Indonesian adolescents with variation in the validity of self-reporting of these oral conditions measured by sensitivity, specificity, LR+ , LR− and AUC. Self-reporting underestimated the presence of caries and overestimated tooth loss and fillings. Specificity was greater than sensitivity and only the LR+ of self-reported presence of filling indicated large change in the probability of the clinically assessed condition.

This study showed that self-reporting underestimated the clinical presence of dental caries. Studies have reported disparities between clinical and self-reported oral health [9] and this may be attributed to poor understanding of oral diseases and their symptoms. Moreover, factors such as gender and socioeconomic status were associated with self-reported oral health [3, 8, 9], while clinical examination might be less likely associated with confounding factors. Our findings are similar to those of previous studies where an underestimation of the number of teeth with caries was self-reported by Brazilian adolescents [3] and Nigerian mothers of young children [4]. This may be due to the participants’ poor awareness of dental caries, as the presence of caries may only be recognized when the lesion affects social relationships or causes pain [4]. Similarly, a study reported no significant association between decayed teeth and impact on daily life in the 12–15-year-old Indonesian adolescents [24]. Thus, caries may not have had a considerable influence on the daily activities of adolescents in the present study and clinical examination may diagnose caries that the person is unaware of [4]. On the other hand, the presence of tooth loss and filling were overestimated, and this may likely be due to the low prevalence of tooth loss and fillings in the study population.

In the present study, the specificity of self-reporting the three conditions was higher than the sensitivity. Sensitivity and specificity must be equally high for self-reporting to be considered accurate [19]. However, sensitivity and specificity are often inversely related [25]. The results of the present study are similar to a previous study conducted in Jakarta, Indonesia, where the specificity of self-reported condition was higher than sensitivity [7, 19]. A screening indicator—such as self-reported presence of caries, fillings, or tooth loss- is desired to have high sensitivity [25] which is not the case for the three indicators in this study. Alternatively, self-reported absence of filled teeth had the highest specificity and thus, this highly specific test can rule in the presence of fillings when it is positive; the SpIn rule [20]. However, further studies are needed to assess the consistency of the present findings in populations with higher prevalence of filled teeth before solid recommendations can be made.

The main findings from this study concern the low accuracy of self-reported results regarding the presence of caries, tooth loss and fillings compared to findings from clinical examinations conducted by calibrated dentist. Data on several parameters related to accuracy (AUC, Sn, Sp) all demonstrate results that do not meet accepted standards or thresholds. The observation that the absolute discrepancies for tooth loss and fillings were less than the discrepancy for caries is likely due to the low prevalence of tooth loss and fillings in the survey participants—not to better performance of the self-reported method. Accordingly, we do not recommend that this method should be used for the assessment of the presence of fillings. Self-reporting caries and tooth loss had minimal to small usefulness in shifting the post probability of the corresponding clinical outcomes and thus they cannot be used instead of clinical examination.

Although the accuracy of self-reported oral health was poor in the present study, alternative self-reporting methods may have practical use in epidemiological settings such as in Indonesia. Self-reporting may serve as an advantageous screening instrument by providing a rapid and inexpensive method to determine those who need referral for further examination [9]. This study has some limitations. First, recall bias may be present due to the long reference period of one year, resulting in participant’s confusion during the interview and fallacious reporting of the oral conditions. Second, the duration of one year recall compared to the time point of oral examination may result in some degree of bias. Some of the assessed conditions may have changed status. For example, caries might have been treated, filled tooth may have become carious, and a carious tooth might have become extracted [26]. Thirds, social desirability bias may be present in responding to questions that address oral condition because they may be considered embarrassing for some participants, such as those assessing the presence of tooth decay, or missing tooth. Thus, the self-reported information given may reflect what the participant thinks is suitable for their image rather than the actual condition. Fourth, the simple tool used in the present study to record self-reported presence of oral conditions might have limited the ability to assess the presence of the conditions. Future studies using different methods of self-reporting oral health are needed. Further studies are also needed for other age groups to analyze self-image differences between age groups.

Previous studies had used various types of survey questions to measure self-reported oral health [1–4, 6–9]. High validity of self-reported condition was observed when the participants were asked a question concerning the number of teeth [9]. Another study used a series of questions to identify adolescents' knowledge of the number of decayed, missing and filled teeth and demonstrated valid self-reporting for population-based health surveys [3]. The use of this type of questionnaire could improve the accuracy of self-reporting oral health in adolescents. Moreover, the reference period in the questionnaire may need to be shortened to three- or six months instead of one year, thus allowing adolescents to have a better memory of their oral in the past and reduce recall bias [26].

Improving the abilities of adolescents to self-report their oral health condition empowers them to assume responsibility for managing their own health [27]. When a person acknowledges the presence of poor oral condition, it helps drive positive health-behavior changes such as performing oral hygiene and regular visits to the dentist, thereby improving oral health [24].

## Conclusion

The study addressed the low accuracy of self-reported oral health to detect caries, tooth loss and fillings clinically assessed by calibrated dentists. Self-reported oral health in Indonesian adolescents has low accuracy compared to clinical examination and should not be used as its substitute.

## Data Availability

The raw data are available from the authors to any author who wishes to collaborate with us.

## Abbreviations

Sn: Sensitivity
Sp: Specificity
LR: Likelihood ratio
AUC: Area under the curve
ROC: Receiver operating characteristic
INOHS: Indonesian National Oral Health Survey
IBHR: Indonesian Basic Health Research
WHO: World Health Organization
CI: Confidence interval
